# The mechanical properties of microbial surfaces and biofilms

**DOI:** 10.1016/j.tcsw.2019.100028

**Published:** 2019-08-05

**Authors:** Glauber R. de S. Araújo, Nathan B. Viana, Fran Gómez, Bruno Pontes, Susana Frases

**Affiliations:** a Laboratório de Ultraestrutura Celular Hertha Meyer, Instituto de Biofísica Carlos Chagas Filho, Universidade Federal do Rio de Janeiro, Rio de Janeiro, RJ, Brazil; b Laboratório de Pinças Óticas (LPO-COPEA), Instituto de Ciências Biomédicas, Universidade Federal do Rio de Janeiro, Rio de Janeiro, RJ, Brazil; c Instituto de Física, Universidade Federal do Rio de Janeiro, Rio de Janeiro, RJ, Brazil; d Centro Nacional de Biologia Estrutural e Bioimagem (CENABIO), Universidade Federal do Rio de Janeiro, Rio de Janeiro, RJ, Brazil

**Keywords:** Rheology, Mechanics, Biofilm, Secreted polysaccharides

## Abstract

Microbes can modify their surface structure as an adaptive mechanism for survival and dissemination in the environment or inside the host. Altering their ability to respond to mechanical stimuli is part of this adaptive process. Since the 1990s, powerful micromanipulation tools have been developed that allow mechanical studies of microbial cell surfaces, exploring little known aspects of their dynamic behavior. This review concentrates on the study of mechanical and rheological properties of bacteria and fungi, focusing on their cell surface dynamics and biofilm formation.

## 1 Introduction

Microorganisms are able to survive in a diversity of environments, including mammalian hosts (Bleuven and Landry, 2016, Lenski et al., 1991, Levins, 1968, Meyers and Bull, 2002). From bacteria to eukaryotes, survival depends on the capacity of a living organism to react to environmental pressures that involve a range of mechanical forces (Weaver, 2017). Recently, a number of studies have examined the role of mechanics in controlling elementary eukaryotic cellular processes including chromatin organization, cell division, protein trafficking, cell adhesion and membrane modifications (Fritton and Weinbaum, 2009, Pontes et al., 2017, Pruitt et al., 2014). As explained by Paul A. Janmey and Manfred Schliwa, “*cells are mechanical as well as chemical and electrical devices, and understanding their biology requires knowledge of all these aspects*” (Janmey and Schliwa, 2008). The response of living cells to the mechanical forces of the environment has critical effects on their ability to grow, divide, differentiate, survive and adhere to surfaces (Ayala et al., 2016, Nussenzveig, 2018, Pontes et al., 2017).

While fungi and bacteria are able to modify their cell shape and surface structure as an adaptive mechanism for survival and dissemination in the environment or inside the host; little is known about the mechanical dynamics of these microbes particularly with respect to their cell surfaces, the outermost region and the most susceptible to external forces.

Rheology is an area of physics that characterizes the elastic and viscous responses of materials when stimulated by external forces. In recent years, different studies have characterized the rheological properties of microorganisms’ cell surfaces. This review provides an overview of studies on the mechanical properties of bacterial and fungal surfaces, focusing on cell wall dynamics, biofilm formation and also highlighting the advances in our understanding of the complex dynamic behavior of these key biological interfaces.

## 2 Rheological models for the study of microbial surfaces

### 2.1 Physical principles and models in rheology

Rheology studies the deformation and flow of materials when subjected to external loads. When a load is exerted on a material, the resulting deformation depends on the material’s properties. Two general deformation behaviors can be observed: (1) reversible elastic deformations that store potential energy, or (2) irreversible viscous/plastic deformations that dissipate energy. The material’s response is characterized by some constitutive equations, known as the rheological equations of state, that are independent of the geometry of the material and usually relate applied stresses to observed strains (Vadillo-Rodríguez and Dutcher, 2011).

In this review we consider shear stresses and strains, without specifying the tensorial character of these physical quantities. Consider the application of a stress or strain to a body results in internal material rearrangements that require a finite and single relaxation time $\tau_{m}$ (material characteristic time scale) to reach a new state of equilibrium, and the material’s response to an imposed stress or strain happens in a time scale $\tau_{\exp}$ (experimental characteristic time scale). If $\tau_{m}\ll \tau_{\exp}$, the material is considered purely viscous and all the energy required to produce the deformation is dissipated as heat. This can be characterized by a viscosity parameter (η) that relates stress to strain rates (Boal, 2012). If the material’s response to an imposed stress or strain happens in a time scale $\tau_{\exp}$ so that $\tau_{m}\gg \tau_{\exp}$, the material is considered purely elastic, and all the energy required to produce the deformation is reversibly stored. This can be characterized by the material’s Young modulus (E), a parameter that relates applied stresses to resulting strain (Boal, 2012). In fact, all biological materials are viscoelastic and exhibit an intermediate behavior between the two extremes described above, in which some energy is stored and some dissipated. At the limit of very small deformations (infinitesimal), the viscoelastic constitutive equation of a material is described by linear differential equations with constant coefficients. The linear viscoelastic behavior of a material is characterized by the Boltzmann superposition principle, which states that the stress (σ) at a given time t, under an arbitrary strain history, is a linear superposition of all strains (ε) applied at previous times multiplied by the values of a weighting function $G\left(t\right)$, (the material shear relaxation modulus) as shown below:

(1) $$\sigma \left(t\right)=\int_{0}^{t}G\left(t-u\right)\varepsilon \left(u\right)du.$$

Using the Fourier analysis formalism, we find that:

(2) $$\overset{\;}{\sigma }\left(\omega \right)=G^{*}\left(\omega \right)\overset{\;}{∊}\left(\omega \right),$$

where $\overset{\;}{\sigma }\left(\omega \right)$ and $\overset{\;}{\varepsilon }\left(\omega \right)$ are, respectively, the Fourier transforms of the stress and strain, ω is the angular frequency of the Fourier signal component and $G^{*}\left(\omega \right)=G^{\text{'}}\left(\omega \right)+iG^{\text{'}\text{'}}\left(\omega \right),$ is the complex shear modulus of the material. The real part of $G^{*}\left(\omega \right)$is the material’s storage modulus and can be interpreted as an approximation of its Young’s modulus E (G=E/3, for incompressible materials) (Boal, 2012). On the other hand, the imaginary part of $G^{*}\left(\omega \right)$is the material’s loss modulus and is related to its viscosity ($\lim_{\omega \to 0}G^{\text{'}\text{'}}\left(\omega \right)/\omega =\eta$,) (Boal, 2012). The linear viscoelastic response of a material is completely characterized by its complex shear modulus, which is usually obtained by analyzing the material’s response to imposed stresses or strains that present a time functional dependence described by sine or cosine functions.

Another way to look at this problem is to consider the applied forces and the resulting deformations that these forces produce in a viscoelastic material. The Fourier transform of the force $\overset{\;}{F}\left(\omega \right)$ is related to the Fourier transform of the deformation $\overset{\;}{x}\left(\omega \right)$ using the formula:

(3) $$\overset{\;}{F}\left(\omega \right)=k^{*}\left(\omega \right)\overset{\;}{x}\left(\omega \right),$$

where $k^{*}\left(\omega \right)=k^{\text{'}}\left(\omega \right)+ik\text{'}\text{'}\left(\omega \right)$ is the complex elastic constant of the material, $k^{*}\left(\omega \right)$ is related to$G^{*}\left(\omega \right)$ by ${k^{*}\left(\omega \right)=G}^{*}\left(\omega \right)\xi$, where ξ has a unit of length and depends on the morphology of the material (Ayala et al., 2016). In this review we discuss elastic and viscoelastic properties from published results of the Young’s modulus, viscosity, complex shear modulus and complex elastic constant that have been measured for various microorganisms, depending on techniques employed (Table 1).

**Table 1:** Summary of mechanical techniques and parameters obtained for a diversity of biomaterials on the surface of microbes.

Material/Sample	Techniques	Models	Parameters	References
*Pseudomonas aeruginosa*	Atomic Force Microscopy and Rheometer	Standard Solid	Spring constants, Viscosity, Relaxation time and Viscoelastic moduli	Vadillo-Rodriguez et al., 2008, Vadillo-Rodriguez et al., 2009, Vadillo-Rodriguez and Dutcher, 2009, Lieleg et al., 2011, Kovach et al., 2017
*Escherichia coli*	Atomic Force Microscopy, Micromanipulation and Langmuir balance	Standard Solid	Spring constant, Young’s Modulus, Bursting forces, Spring constants, Viscosity and Relaxation time	Shiu et al., 1999, Vadillo-Rodriguez et al., 2008, Vadillo-Rodriguez et al., 2009, Vadillo-Rodriguez and Dutcher, 2009, Cerf et al., 2009, López-Montero et al., 2012, López-Montero et al., 2013
*Shewanella putrefaciens*	Atomic Force Microscopy		Spring constant	Gaboriaud et al. (2005)
*Shewanella oneidensis*	Atomic Force Microscopy		Young’s modulus and Spring constant	Gaboriaud et al. (2008)
*Staphylococcus aureus*	Atomic Force Microscopy	Kelvin-Voigt	Young’s modulus, Spring constant, Viscosity and Relatation time	Eaton et al. (2008)
*Bacillus subtilis*	Atomic Force Microscopy	Standard Solid	Spring constants, Viscosity and Relaxation time	Vadillo-Rodriguez et al., 2008, Vadillo-Rodriguez et al., 2009, Vadillo-Rodriguez and Dutcher, 2009
*Termitomyces clypeatus*	Atomic Force Microscopy	Kelvin-Voigt	Young’s modulus and Spring constant	Das et al. (2009)
*Aspergillus nidulants*	Atomic Force Microscopy		Young’s modulus, Spring constant, Force cell wall, Relative rigidity, Relativity adhesion and Cell wall viscoelastic moduli	Ma et al., 2005, Paul et al., 2011
*Staphylococcus epidermis*	Atomic Force Microscopy, Micromanipulation and Rheometry	Kelvin-Voigt	Young’s modulus, Viscosity, Relaxation time, Bursting forces and Viscoelastic moduli	Das et al., 2009, Pavlovsky et al., 2013
*Staphylococcus salivarus*	Atomic Force Microscopy	Kelvin-Voigt	Young’s modulus, Viscosity and Relaxation time	Das et al. (2009)
*Cryptococcus* spp. (Polysaccharide capsule)	Dynamic light-scattering and Optical tweezers		Young’s modulus, Zeta potential and Size determination	Nimrichter et al., 2007, Frases et al., 2009a, Frases et al., 2009b, Cordero et al., 2011, Cordero et al., 2011, de Araujo et al., 2012, Cordero et al., 2013, Cordero et al., 2013, Albuquerque et al., 2014, Pontes and Frases, 2015
*Streptococcus* spp.	Atomic Force Microscopy	Kelvin-Voigt	Young’s modulus and Viscosity	Chen et al. (2014)
*Streptococcus mutans*	Rheometer (Constant shear stresses)		Viscoelastic moduli	Vinogradov et al. (2004)
Biofilms of *Bacillus subtilis, Staphylococcus aureus* and *Pseudomonas aeruginosa*	Rheology and Passive Microrheology Techniques		Permeability, Mechanical properties of the biofilm matrix and its interacting components	Billings et al. (2015)

**Fig. 1:**
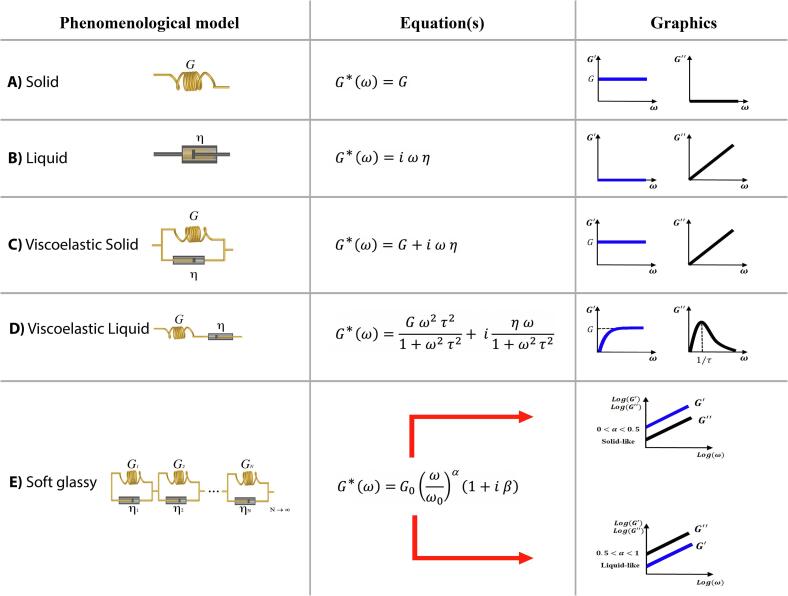
Phenomenological models for rheological analysis. The symbols represent (A) the solid (spring, shear modulus G) and (B) liquid (dashpot, viscosity η) behaviors, and the plots of their dependencies with angular frequency. The spring characterizes energy storage in the material, and the relationship between stress and strain is given by σ=G∊. The dashpot characterizes the energy loss in the material and the relationship between stress and strain is given by $\sigma =\eta \frac{d∊}{\mathrm{dt}}.$ The association of one spring and one dashpot in parallel gives rise to the Kelvin-Voigt model for a viscoelastic solid (C). The association in series represents the Maxwell model for a viscoelastic liquid (D). Both the Maxwell and Kelvin-Voigt models present only one relaxation time, τ=η/G. The combination of an infinite number of Kelvin-Voigt blocks describes the model for soft glassy materials (E), characterized by a power law dependency of the complex shear modulus with the angular frequency. The power law dependency is also a signature of infinity time-scales present in the response. When $G^{\text{'}}>G\text{'}\text{'}$, a solid-like behavior is observed. When $G^{\text{'}}<G\text{'}\text{'}$ a liquid-like behavior is visualized.

Fig. 1 presents a summary of the simplest elastic and viscoelastic phenomenological models used to describe materials responses. The equations used for the complex shear modulus as well as the plots of their dependencies with the angular frequency are also shown. One spring (Fig. 1A, a spring represents the elastic behavior of a solid material, since for this case the material strain is proportional to the applied stress) and one dashpot (Fig. 1B, a dashpot represents the behavior of a Newtonian liquid, since for this case the rate of strain in the material is proportional to the applied stress). The association of one spring and one dashpot in parallel gives rise to the Kelvin-Voigt model for a viscoelastic solid (Fig. 1C). The association in series represents the Maxwell model for a viscoelastic liquid (Fig. 1D). Both the Maxwell and Kelvin-Voigt models present only one relaxation time, τ=η/G. The combination of a infinite number of Kelvin-Voigt blocks describes the model for soft glassy materials (Balland et al., 2006) (Fig. 1E), characterized by a power law dependency of the complex shear modulus with the angular frequency. The power law dependency is also a signature of infinity time-scales present in the response. When $G^{\text{'}}>G\text{'}\text{'}$, a solid-like behavior is observed. When $G^{\text{'}}<G\text{'}\text{'}$ a liquid-like behavior is visualized.

## 3 Complex shear modulus measurements

Soft glassy materials, such as the bacterial envelope, bacterial biofilms, fungal capsules and the cell membrane, are deformable. The advent of modern micromanipulation tools opened up a myriad of possibilities to describe the mechanical properties of these biological materials. These tools include optical tweezers (Prescott et al., 2008), magnetic tweezers (Doyle and Marquis, 1994), atomic force spectroscopy (AFS) (Whatmore and Reed, 1990), micropipette aspiration (Hochmuth, 2000), microplate rheometer (Fernández and Ott, 2008), particle-tracking microrheology (Wirtz, 2009) and the development of dynamic light scattering (DLS) to characterize viscoelastic solutions (Mason, 2000).

Using these micromanipulation tools, viscoelastic properties could be determined by applying forces and measuring displacements as functions of the imposed load. This procedure, known as active micromechanical measurements, enables the determination of the material’s Young’s modulus or the complex shear modulus by analyzing the material’s deformation in response to an imposed stress. Active micromechanical measurements can be performed using AFM, optical tweezers and/or magnetic tweezers. The range of frequencies explored goes up to hundreds of hertz. The strains used in active micromechanical measurements experiments can exceed the limit of linear responses, allowing the non-linear (as well as the linear) regime of the material’s viscoelasticity to be observed (Mason, 2000).

With particle-tracking microrheology and/or DLS, the analysis of the Brownian motion of a spherical probe (μmin diameter) in a complex material is used to characterize the material’s viscoelastic properties. These passive micromechanical measurements allow the complex shear modulus of a given material to be determined for a much wider frequency range than in active micromechanical measurements (reaching kilohertz values). The technique essentially characterizes linear responses (Mason and Weitz, 1995).

The mechanical properties of bacterial and fungal surfaces contribute a great deal to microbial function. These physical parameters have been analyzed using several nanoscale techniques. A brief summary containing the different techniques used, together with the parameters obtained for a diversity of microbes is highlighted in Table 1, some of these results were already discussed in this review.

## 4 Micromechanical properties applied to the bacterial envelope and the fungal cell wall

Bacteria and fungi cells are encased in a cell wall that protects them from the surrounding environment. In addition to controlling the exchange of substances with the outside environment, the cell wall not only participates in cell growth but also acts as a physical rigid barrier that mechanically protects these cells against various external forces. Therefore, understanding how cells walls react to external forces will help to better elucidate how bacteria and fungi survived billions of years under extreme environmental conditions by characterizing the responses of these microorganism to controlled external forces.

In the 1960s, the tools required to deform bacterial and fungal cells in a controlled fashion were not available, making the first experiments purely qualitative by evaluating which elements of the cell wall influenced its flexibility (Marquis, 1968). Using the model bacterium Escherichia Coli, Koch and Woeste (1984) demonstrated that its Gram-negative envelope expand up to three times its length in comparison to a relaxed state, while its surface area decreased by 20% with changes to medium pH (Koch, 1984, Koch and Woeste, 1992).

In the 1990s, the challenge of deforming living cells was overcome by the emergence of optical tweezers, magnetic tweezers, atomic force spectroscopy (AFS) and other technical advances that offered the possibility of individual cell manipulation. These techniques enabled the measurement of the deformation produced on a certain area of ​​the cell, and an estimate of Young’s modulus and viscoelastic properties (Marquis, 1968).

One of the first historical studies on the mechanical properties of single bacterial cells dated back from the late 1990s (Shiu et al., 1999). *Escherichia Coli* bacterial cells of both Gram-positive and Gram-negative strains were compressed between a glass coverslip and an optical fiber which was attached to a force transducer, allowing measurements of the force required to rupture the bacteria. The average forces measured were 13.8 μN and 3.6 μN, respectively, for *E. coli* Gram-positive and Gram-negative cells (Shiu et al., 1999). With the advent of sophisticated atomic force microscopes, enabling force spectroscopy, researchers were able to better characterize the elastic properties (Young’s modulus) of bacterial envelopes, with reported values ranging from 10^7^ to 10^8^ Pa, depending on the bacterial strain used (Cerf et al., 2009, Eaton et al., 2008, Gaboriaud et al., 2008, Gaboriaud et al., 2005).

While the Young’s modulus has been the selected parameter to characterize the mechanical properties of bacterial cell envelopes, it does not provide a complete description, since bacterial envelopes display not only elastic but also viscous behaviors (Vadillo-Rodríguez and Dutcher, 2011). To provide a more complete description of the mechanical properties of bacterial envelopes, a new method was developed using a colloidal tip instead of a sharp AFS tip, and applied to bacterial cells. The colloidal tip was used to indent bacterial envelopes and the viscoelastic parameters determined for different bacterial strains (Vadillo-Rodriguez et al., 2009, Vadillo-Rodriguez et al., 2008, Vadillo-Rodriguez and Dutcher, 2009). This new setup was able to identify, for example, clear differences in the viscoelastic properties between Gram-negative and Gram-positive cells (Vadillo-Rodriguez et al., 2009). Moreover, it also showed that the elastic component was dominated by the peptidoglycans in the bacterial envelope, whereas the viscous component reflects the liquid-like behavior of the membranes of the bacterial envelopes (Vadillo-Rodriguez et al., 2009, Vadillo-Rodriguez and Dutcher, 2009). The viscoelastic properties not only vary with the biochemical nature of the envelope but also depend on the conditions to which the bacteria are subjected. For example, the degree of hydration completely changes the properties of the envelope (Vadillo-Rodriguez et al., 2009) and, during cell division, FtsZ polymerization – depolymerization cycles increase or decrease fluidity, respectively, which alters the viscoelasticity of the envelope (López-Montero et al., 2013, López-Montero et al., 2012).

Apart from bacteria, the mechanical properties of fungal cells are mainly dependent on their cell wall, which contains four major components: β-(1,3)-glucan, β-(1,6)-glucan, chitin (N-acetylglucosamine) and glycoproteins, with the exact composition varying between species. In *Candida albicans*, for example, the outer cell wall is enriched with a fibrillar layer of highly glycosylated mannoproteins (Hall and Gow, 2013). *Aspergillus fumigatus* cell wall has fewer proteins, but includes two bioactive polysaccharides, galactomannan and galactosaminoglycan. On the other hand, in *Cryptococcus* spp. the outer wall is surrounded by a thick capsule composed of glucuronoxylomannan and galactoxylomannan (Walker et al., 2018). Moreover, some fungi can exist as unicellular yeasts or as hyphae; so, in some cases thus, it is important to analyze the mechanical properties of cell walls both in hyphae (filamentous fungi) and in yeasts (unicellular fungi).

Only a limited number of studies have attempted the challenging task of determining the mechanical properties of fungal cell walls. AFM-based measurements showed that the viscoelasticity of the hyphal wall of *Aspergillus nidulans* changes depending on its composition*.* Immature hyphal wall regions or those devoid of β-galactofuranose have lower elastic parameters than mature and wild type (β-galactofuranose-containing) areas, respectively (Ma et al., 2005, Paul et al., 2011).

AFM-based measurements in *Termitomyces clypeatus,* a Basidiomycete*,* showed that the cell wall rigidity and elastic properties increase when the organism reaches the stationary phase in culture, with a sudden decrease in these mechanical parameters at the onset of the death phase (Das et al., 2009).

Optical Tweezer based methods have also emerged as powerful tools to study the mechanics of the *Cryptococcus* spp*.* capsule (setup reviewed, in (Pontes and Frases, 2015). The elastic properties (characterized by the ‘Young’s modulus’) of the *C. neoformans* polysaccharide capsule can be accurately and reproducibly measured under several conditions for live cells (Cordero et al., 2011, Cordero et al., 2013, de Araujo et al., 2012, Frases et al., 2009a, Frases et al., 2009b). A comparison between the elastic properties of pathogenic and non-pathogenic *Cryptococcus* species showed an increase of up to 2.5-fold in the elastic properties of the capsule of non-pathogenic *C. liquefaciens* (de Araujo et al., 2012), showing that pathogenic *C. neoformans* species have softer capsules. Taken together the data suggests that the capsular elastic properties may represent a key mechanical component linked to the pathogenicity of encapsulated fungi. The elastic properties of the *C. neoformans* capsule increased as a function of the concentration of divalent ions, such as Ca^2+^ (Frases et al., 2009a, Frases et al., 2009b), which contribute to the self-aggregation of polysaccharide fibers, possibly by forming intra- and/or intermolecular links between (or within) them (Nimrichter et al., 2007). The elastic properties of the *C. neoformans* capsule also change as a function of antibody binding. The binding of protective (but not that of non-protective) antibodies produces a concentration-dependent increase in capsule stiffness, likely due to antibody-mediated cross-linking of polysaccharides molecules. This effect may result in the formation of a “sac-like” structure (derived from the parental cell’s capsule) that traps and prevents the release of daughter cells, thereby reducing pathogen dissemination and increasing the chances of pathogen phagocytosis during infection (Cordero et al., 2013).

Young’s modulus only describes the elastic behavior of the capsule. A new optical tweezers-based micromechanical measurement technique is being developed to examine both the elastic and the viscous behavior of the capsule. Indeed, the viscosity of isolated polysaccharides has already been described using optical tweezer based techniques or viscometers (Albuquerque et al., 2014, Frases et al., 2009a, Frases et al., 2009b, Rodrigues et al., 2007). However, to the best of our knowledge, no study so far has performed measurements of the capsule’s viscoelastic behavior in its native state. This new experimental setup should provide a more complete picture of the global mechanical parameters (elastic and viscous) of the capsule (de Araujo et al., 2019).

## 5 Extracellular matrix and microbial biofilm

The term “biofilm” is defined as a community of microorganisms that secrete and grow embedded in an extracellular matrix (ECM) while adhered to an inert surface or a living tissue. Recent, multidisciplinary studies have shown that bacteria and fungi in most biological systems exist as biofilms, rather than in a free-living state, and that microbes behave very differently in biofilms, compared with planktonic growth (Gabrilska and Rumbaugh, 2015). Biofilms have features of both solids and liquids, their real mechanical response to forces resembles that of a viscoelastic fluid (Wilking et al., 2011).

Biofilms are highly organized microbial communities. The ability of pathogens to form biofilms plays a significant role in their virulence as well as in conferring resistance to antimicrobials. Bacteria and fungi capable of producing biofilms secrete surface adhesion molecules that form an ECM (Costa-Orlandi et al., 2017, Sheppard and Howell, 2016, Visick et al., 2016).

Biofilm dynamics are controlled by the environment, which influences its structural, physical, mechanical and chemical properties (Billings et al., 2015), as well as the interactions between resident microbes.

**Fig. 2:**
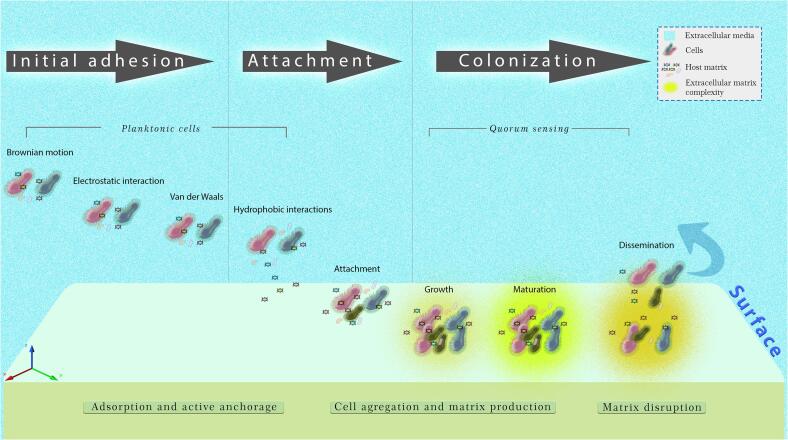
Stages present during the development of a biofilm on a substrate. The formation of biofilms occurs as a series of sequential events that depend on the interaction of microorganisms on inert or living surfaces, overcoming the forces of repulsion until achieving irreversible adsorption followed by the formation of a microcolony. When reaching a certain population density, the synthesis of secondary metabolites (quorum sensing) is induced, which produces the formation of an exopolysaccharide until the maturation of the biofilm is achieved. Disintegration allows the formation of a new colony or its elimination.

Biofilm formation follows a sequence of phases (Fig. 2). The first stage represents the interaction of planktonic cells with a surface, which is influenced by the substrate’s chemical structure and topography (exposed functional groups, surface charge, hydrophobicity, roughness, etc.), by environmental, nutritional and physical conditions (pH, temperature, pressure, liquid flow velocity, shear force), as well as by the presence of other microorganisms (Costa-Orlandi et al., 2017, Sheppard and Howell, 2016, Visick et al., 2016). The second and irreversible phase of biofilm formation is its attachment to a surface (Fig. 2). In the third phase, microbes begin to secrete substances that will be responsible for maintaining strong adhesions and forming the ECM layer that surrounds the biofilm (Fig. 2). At this stage, the formation of microcolonies and the development of a mature biofilm architecture begin. Mature biofilms have a complex structure, where cells are surrounded by several substances (mainly sugars), as well as by pores and water channels that function as a system for the passage of nutrients, oxygen and metabolites destined for secretion. In the final stage, the biofilm cannot be maintained, and thus, it is released either as planktonic cellular or cell aggregates (biofilm detachment). Subsequently, free microbes can colonize new environments, establishing new biofilms (Costa-Orlandi et al., 2017, Sheppard and Howell, 2016, Visick et al., 2016).

Biofilms have been subjected to various mechanical challenges, in attempts to better elucidate their mechanical properties at different stages of development. Most studies in which biofilms were subjected to mechanical or chemical injuries focused on the stiffness and viscoelasticity of bacterial biofilms, in pathogenic strains of *Staphylococccus, Pseudomonas, Bacillus* and *Streptococcus* (Lieleg et al., 2011, Wang et al., 2018). Lieleg and co-workers examined several Pseudomonas biofilms and did not observe an effect of antibiotics on biofilm mechanical properties.

Certain parts of the biofilms capitalize on applied force to persist in the environment, by remaining attached and merging into surfaces, while other parts of the biofilm detach in response to external mechanical forces (Lieleg et al., 2011). The viscoelastic properties of *Pseudomonas aeruginosa* biofilms are resistant to chemical treatment and strong shear forces, allow them to efficiently recover from mechanical damage. One implication of differential response to force is that fragments of biofilms could merge with adjacent biofilm sections, thereby spreading (rather than removing) the biofilm. These processes play an important function in infection persistence (Dunsmore et al., 2002, Lieleg et al., 2011, Stoodley et al., 2002).

Bacterial sensing affects the mechanical properties of mature biofilms (Kovach et al., 2017). The impact of the shear stress under which biofilms are formed affects their sensitivity to chemical and mechanical stresses (Kovach et al., 2017). *Bacillus cereus* biofilms can form under varying hydrodynamic conditions, associated with different shear stresses. Increasing the shear stress during biofilm formation resulted in biofilms with lower thickness, higher dry mass and higher volumetric and cell densities. Although biofilms formed under low shear stress were more resistant to removal by chemical treatment, biofilms formed under higher shear stress were more resistant and the combination of chemical and mechanical treatments (Lemos et al., 2015). For several bacterial species, biofilms that were initiated and grown under conditions of high shear are stiffer and denser in proteins and polysaccharides than biofilms grown under low shear (Kovach et al., 2017, Lemos et al., 2015)

Secreted bacterial polysaccharides perform an important function in biofilm ecology, playing a role in signaling and quorum sensing (Fujishige et al., 2008, Rinaudi et al., 2010, Wang et al., 2008). Despite their importance their polymeric rheological properties remain understudied. The rheological properties of the secreted polysaccharides and biofilm ECM are influenced by the availability of water in the environment where the biofilm develops. Although *B. subtilis* biofilms could be found in dehydrated material (catheters polymers such as silicone rubber, nylon, polyurethane) inside the host, *P. aeruginosa* biofilms formed in catheters grow in direct contact with aqueous liquids and their water content is high. Rheological measurements showed that biofilms grown in media with low water content have higher elastic modulus than those grown in direct contact with a water source (Wilking et al., 2011). Although not yet demonstrated, elasticity of biofilm could be related to their persistence or resilience or another important factor essential for spreading infection.

The dynamic viscosity of *Streptococcus mutans* biofilms in dental plaque decreases in response to increasing oscillation frequencies, as observed in other biological fluids (such as saliva and mucus) (Vinogradov et al., 2004). While biofilm formation is beneficial in certain settings, such as in waste water treatment, they can be dangerous inside patients, contaminating medical implants and leading to sepsis, or aggravating medical conditions such as cystic fibrosis (Hall-Stoodley et al., 2004). While the rheology of whole biofilms formed in waste water has been characterized (Pavlovsky et al., 2013), the specific contribution of extracellular polysaccharides to bulk biofilm rheology has not been elucidated.

Fungal biofilms have essentially the same functions as their bacterial counterparts, but the rheological behavior of fungal biofilms is less well studied than that of bacterial biofilms. The mechanical properties of biofilms from *Rhodotorula mucilaginosa*, *Candida krusei*, *Candida kefyr* and *Candida tropicalis* were examined with a focus on industrial applications. Essentially, yeast biofilms of these species were determined to be viscoelastic materials with a solid-like behavior (Brugnoni et al., 2014, Tarifa et al., 2017).

The growth of pathogenic microorganisms in biofilms makes their eradication difficult, since they represent an adaptation with improved persistence in the host. Thus, future studies examining the mechanical properties of biofilms from clinical isolates will aid in the development of new strategies to combat infections and prevent pathogens from colonizing medical devices inside patients.

## 6 Conclusions

The mechanical properties of bacterial and fungal surfaces are extremely important to future multidisciplinary studies correlating surface mechanics with bacterial and fungal survival, and will provide novel information towards developing new innovative strategies against pathogens. When the mechanical studies are taken together with classic biochemical descriptions, there is a tremendous potential to better elucidate unanswered microbiological questions.

## Declaration of Competing Interest

The authors declare that they have no known competing financial interests or personal relationships that could have appeared to influence the work reported in this paper.
